# A high performance lithium-ion–sulfur battery with a free-standing carbon matrix supported Li-rich alloy anode†
†Electronic supplementary information (ESI) available. See DOI: 10.1039/c8sc02897d


**DOI:** 10.1039/c8sc02897d

**Published:** 2018-09-24

**Authors:** Tao Zhang, Min Hong, Jun Yang, Zhixin Xu, Jiulin Wang, Yongsheng Guo, Chengdu Liang

**Affiliations:** a Shanghai Electrochemical Energy Devices Research Center , School of Chemistry and Chemical Engineering , Shanghai Jiao Tong University , Shanghai 200240 , P. R. China . Email: yangj723@sjtu.edu.cn ; Email: wangjiulin@sjtu.edu.cn; b Department of Micro/Nano Electronics , School of Electronic Information and Electrical Engineering , Shanghai Jiao Tong University , Shanghai 200240 , P. R. China; c Research Institute , Ningde Contemporary Amperex Technology Co., Limited , Fujian 352100 , P. R. China

## Abstract

Although the lithium–sulfur battery exhibits high capacity and energy density, the cycling performance is severely retarded by dendrite formation and side-reactions of the lithium metal anode and the shuttle effect of polysulfides. Therefore, exploring lithium rich-alloy (or compound) anodes and suppressing the shuttling of polysulfides have become practical technical challenges for the commercialization of lithium–sulfur batteries. Here, a lithium ion sulfur full battery system combining a lithium-rich Li–Si alloy anode and sulfurized polyacrylonitrile (S@pPAN) cathode has been proposed. The free-standing CNF matrix supported Li–Si alloy anode is prepared by a simple and effective method, which is practical for scale-up production. The obtained Li–Si alloy anode demonstrates high cycling stability without dendrite growth, while the use of the S@pPAN cathode avoids the shuttle effect in carbonate electrolytes. The constructed Li–Si/S@pPAN battery could be cycled more than 1000 times at 1C and 3000 times at 3C, with a capacity fading rate of 0.01% and 0.03% per cycle. The exceptional performance should originate from the stable integrated anode structure and the excellent compatibility of the S@pPAN cathode and Li–Si alloy anode with carbonate electrolytes.

## Abstract 2

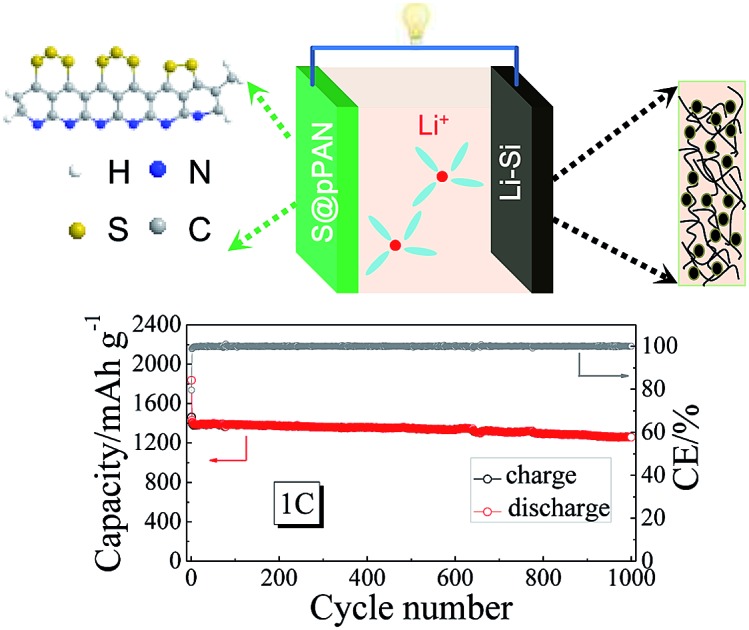
A high-performance lithium-ion–sulfur battery has been built by using a carbon supported Li-rich alloy anode and sulfurized polyacrylonitrile (S@pPAN) cathode.

## Introduction

Lithium–sulfur (Li–S) batteries represent a promising alternative to the state-of-the-art lithium-ion battery (LIB) owing to their high theoretical capacity (1672 mA h g^–1^ for sulfur) and low cost.^1^–^3^ However, the commercial application of Li–S batteries is still retarded by several unfavorable factors including the insufficient utilization of sulfur caused by its electrical insulativity, the large volume change during cycling and the lithium polysulfide shuttling in ether-based electrolytes, which result in capacity fading and poor coulombic efficiency (CE).^4^,^5^ Meanwhile, the metallic Li anode suffers from inhomogeneous deposition and side-reactions during cycling, which could trigger dendrite growth, consumption of electrolyte, low coulombic efficiency and pulverization of the electrode.^6^–^8^

A considerable number of approaches have been developed to tackle these problems. Nanostructured porous carbon,^9^–^13^ metal oxides/sulfides^14^–^21^ and metal–organic-frameworks^22^–^24^ are often adopted for embedding active sulfur and creating conductive networks. Although porous carbon or other host materials possess a high surface area which could partially absorb the lithium polysulfides, more electrolyte is required to wet the large volume of porous carbon. Accordingly, many strategies have been developed to combat lithium polysulfide shuttling, such as introducing interlayers as a sieve,^25^–^27^ modified separators^28^–^30^ and solid state electrolytes.^31^,^32^ However, it is difficult to eliminate the polysulfide shuttle effect substantially using such physical or adsorptive measures, or they bring about other problems. Differing from elemental sulfur based cathode materials, sulfurized polyacrylonitrile (S@pPAN) has shown excellent electrochemical reversibility and no soluble lithium polysulfides in carbonate electrolytes and has been considered as a promising cathode material for Li–S batteries.^33^–^38^

Concerning the practical application of Li–S batteries, the lithium metal anode has become the most critical factor. Extensive work has been carried out to alleviate the dendrite growth and enhance the coulombic efficiency, including utilization of a 3D structured matrix,^39^,^40^ electrolyte additives,^41^–^44^ protective films,^45^–^47^ interfacial engineering^7^,^48^–^50^ and solid state electrolytes.^31^,^51^–^54^ However, these approaches do not fundamentally resolve the problem, and there is still a long way to go to address these obstacles before real applications become possible.

An alternative to metallic lithium is Li-rich materials with enough low delithiation potential, including graphite, Si, Sn, Al and others. Some work has been reported for lithium ion sulfur full cells. Wu *et al.* reported a pre-lithiated graphite and sulfur full cell.^55^ But the difference of specific capacities is quite large for both the electrodes. A lithium ion cell has been proposed by combining a Li_2_S cathode and Sn anode.^56^ Several other groups also studied the compatibility of a sulfur or Li_2_S cathode with Si or Li-based alloy anodes.^57^–^60^ However, the cycling stability of Li-based alloy anodes in ether based electrolytes is still unsatisfactory; thus the full cells could not achieve long-term cycling stability.^60^–^62^ In addition, most of the Li–Si electrodes were obtained by electrochemical lithiation of Si-based electrodes, which is very difficult to employ for practical application. Recently, Al foil partially lithiated on one side has been proposed as an anode to form a lithium ion cell with a S@pPAN cathode.^63^ Although the cell can be cycled well, the coulombic efficiency is low for such an anode (88–94%) because part of inserted lithium will diffuse into the deep Al region and cannot be extracted. Moreover, for a high Al utilization for Li storage, its mechanical degradation or pulverization is unavoidable.

**Fig. 1:**
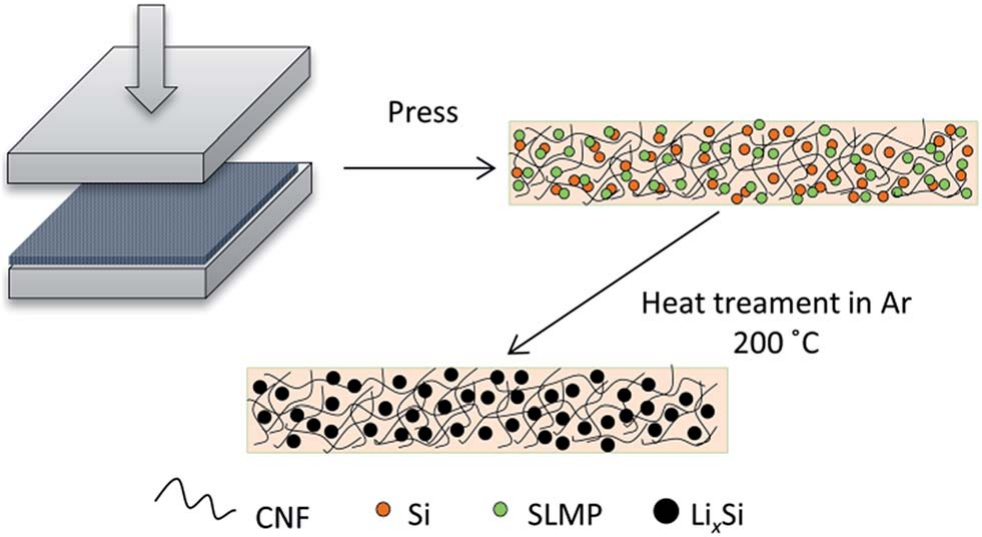
Illustration of the preparation process of carbon supported Li–Si alloy electrodes.

In this work, we have designed a carbon matrix supported Li–Si alloy anode, which is prepared by an easy pressing and heat-treatment process as shown in Fig. 1. When combined with a S@pPAN cathode, the full cell shows excellent long-term cycling performance under high capacity and no dendrite risk. This study pushes the development of high capacity cell systems towards potential application.

## Results and discussion

**Fig. 2:**
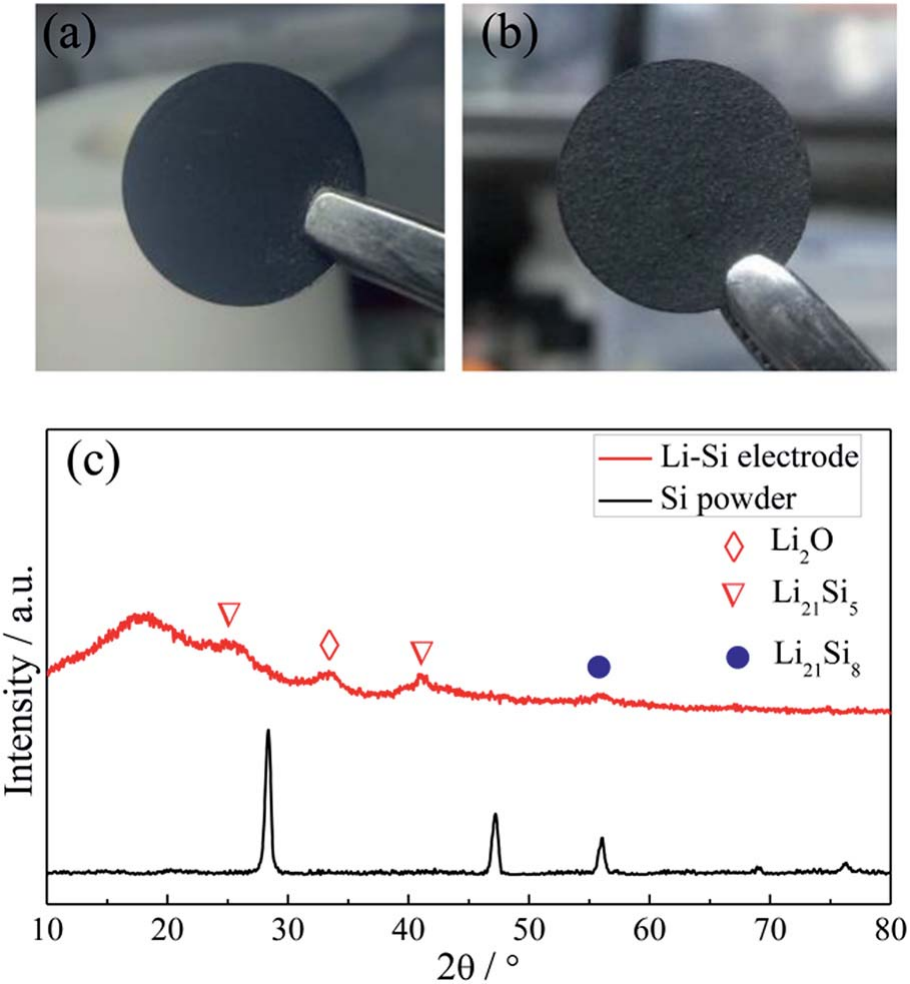
The photo images of a fresh pressed Li–Si–CNF composite (a) and a heat treated Li–Si alloy electrode (b), and the XRD patterns of Si powder and the Li–Si alloy electrode (c).

Fig. 2a and b show the photo images of the pressed Li–Si composite and heat-treated alloy electrodes, which are free-standing with a certain mechanical strength. The heat-treatment made its color slightly lighter. The XRD patterns of Si powder and the heat-treated Li–Si alloy electrode are shown as in Fig. 2c, in which the characteristic peaks of Si disappeared after heat treatment. The main peaks of the Li–Si alloy electrode could be indexed to a Li_21_Si_5_ and Li_21_Si_8_ phase. A small peak located at ∼33° indicates a small amount of Li_2_O. Fig. S1† shows the morphologies of stable lithium metal powder (SLMP), Si powder and CNF. The SLMP and Si powders possess a spherical particle shape with diameters of 20–40 μm and ∼50 nm, respectively. The CNF shows a diameter of 50–70 nm. The initial discharge capacity of CNF is ∼800 mA h g^–1^ and the stable capacity is ∼150 mA h g^–1^ (Fig. S2†). Thus the weight ratio of Si to SLMP was optimized to 1.5 : 1 to compensate for the Li loss during the heating process and irreversible capacity of CNF.

**Fig. 3:**
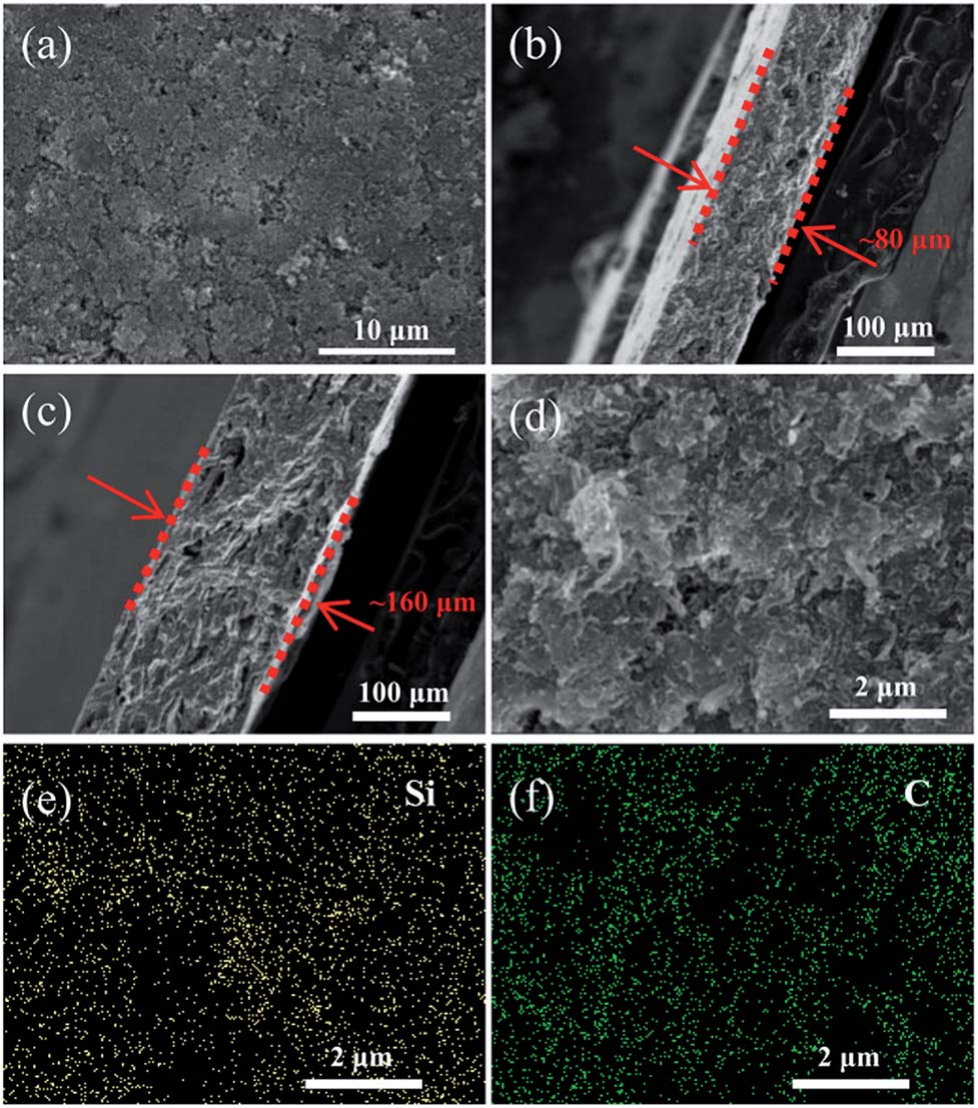
The SEM images of the surface (a) and cross-sectional view (b and c) of the Li–Si alloy electrodes; magnified SEM image of the cross-sectional view (d) and elemental distribution of Si (e) and C (f) by EDS.

As shown in Fig. 3a, the surface of the heat-treated Li–Si electrode (60 wt% Li–Si) is even with some pores. The cross-sectional view images of the Li–Si alloy electrodes indicate the existence of many small holes (or voids) inside the electrodes (Fig. 3b–c). The thicknesses of the electrodes are ∼80 μm and ∼160 μm, respectively, corresponding to different areal capacities. Fig. 3d–f show that the Li–Si alloy particles are embedded homogeneously in the CNF matrix. The X-ray photoelectron spectrum (XPS) of the Li–Si electrode in Fig. S3a† identifies C, O and Li on the electrode surface, but without any Si peak (Fig. S3b†). This could be attributed to the oxidized layer on the surface, including Li_2_O and Li_2_CO_3_ (∼290 eV) as indicated in Fig. S3c.† For the Li–Si electrode without heat treatment, violent side-reactions took place as the electrode was brought into contact with the electrolyte due to the large surface area and high reactivity without sufficient surface passivation from the above oxidized layer.

**Fig. 4:**
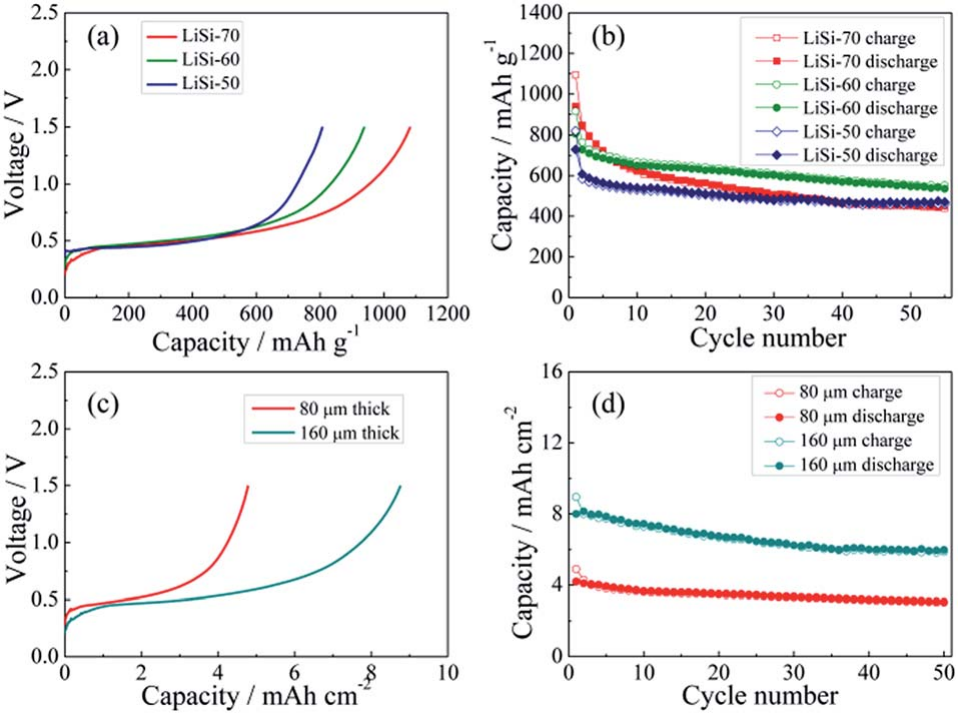
The initial charge curves (a) and cycling performance (b) of the Li–Si alloy electrode with 50, 60 and 70 wt% Li–Si (0.01–1.5 V, 100 mA g^–1^); the initial charge curves (c) and cycling performance (d) of the Li–Si alloy electrode containing 60 wt% Li–Si with different thicknesses (0.01–1.5 V, 100 mA g^–1^).

The electrochemical performance of the Li–Si electrodes was tested with half cells using Li metal as the counter electrode. The initial charging curves of the Li–Si electrodes are shown in Fig. 4a. The initial charging capacity could represent the Li storage capacity of the electrodes. The three electrodes with different Li–Si alloy contents are charged to 1.5 V at 100 mA g^–1^ and all electrodes show a long plateau at ∼0.4 V, and the capacities calculated from the total weight of the electrodes are 807, 938 and 1082 mA h g^–1^, corresponding to 50, 60 and 70 wt% alloy content. Fig. 4b shows the cycling performance of the alloy electrodes under near 100% DOD, where the electrodes containing 50% and 60% alloy possess good cycling performance and the capacity retentions after 55 cycles are 77% and 75%, respectively. Although the electrode containing 70% alloy delivers a higher initial charging capacity, its cycling performance is inferior. This might be ascribed to the instability of the electrode structure. When the alloy content is larger than 70%, a stable free-standing electrode cannot be fabricated. To balance both the capacity and cycling stability, the electrode with 60% alloy is adopted for further investigation. As shown in Fig. 4c, the areal capacities of the alloy electrodes with thicknesses of 80 μm and 160 μm are ∼4.5 and ∼9 mA h cm^–2^, respectively. The thinner electrode presents slightly better cycling stability (Fig. 4d). In addition, the cycling behavior of the Li–Si electrode (60 wt% alloy and a total capacity of ∼4.5 mA h cm^–2^) under a limited capacity of 1 mA h cm^–2^ has also been examined. As shown in Fig. S4,† the quite stable charge and discharge voltage trends in the range of 0.1–0.4 V indicate the high reversibility of lithiation and delithiation.

**Fig. 5:**
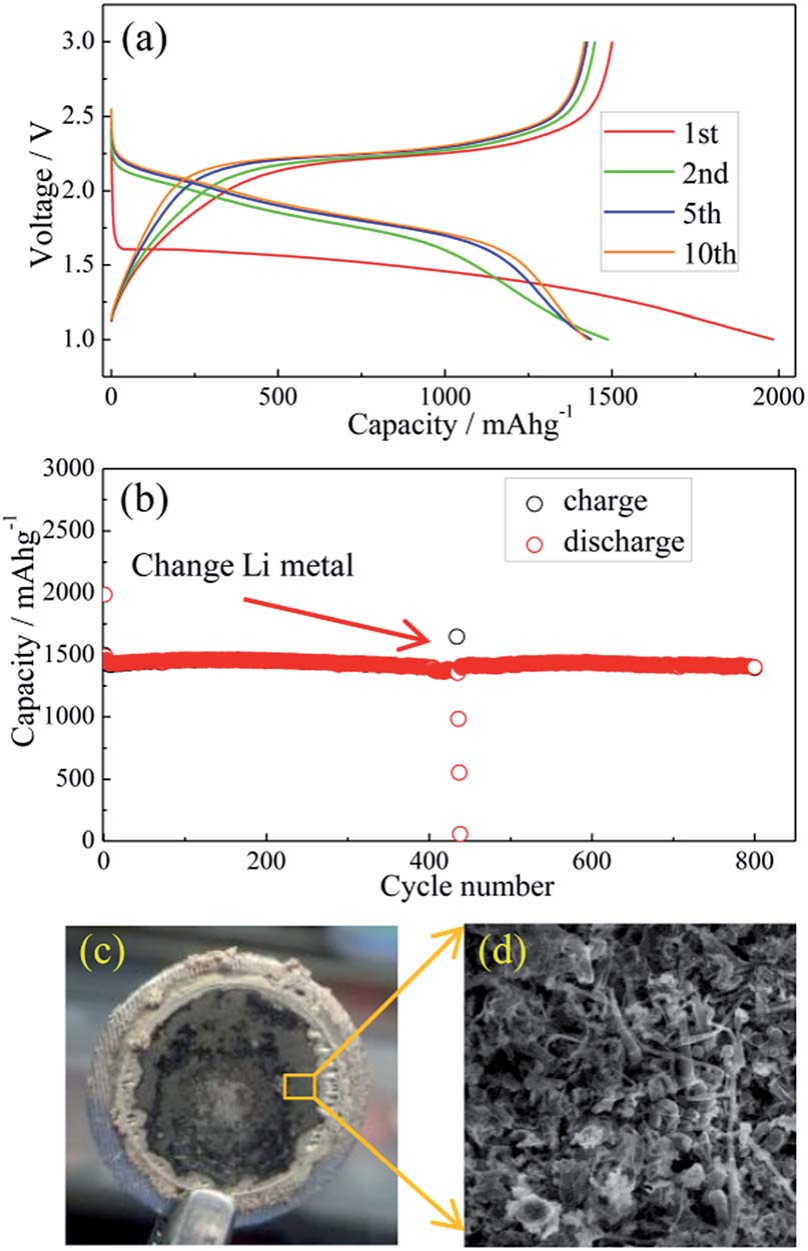
The charge/discharge curves of the Li/S@pPAN cell from 1 V to 3 V at 1C (1C = 1675 mA g^–1^) (a) and its cycling performance (b); the photo image (c) and SEM image (d) of the Li metal anode after 435 cycles of the Li–S@pPAN cell.

Li metal and Li–Si alloy anodes have been coupled respectively with a S@pPAN cathode to form coin cells and their cycling behaviors have been evaluated. As shown in Fig. 5a, the initial discharge/charge capacities of the Li/S@pPAN cell are 1985 mA h g^–1^ and 1496 mA h g^–1^, respectively, indicating a high utilization of sulfur. The first discharge plateau is at ∼1.5 V, but the voltage plateau gradually shifts upward to ∼2.0 V in the following cycles after the initial S@pPAN activation. Fig. 5b shows that the Li/S@pPAN cell could cycle more than 400 times without capacity decay, indicating the ultrahigh stability of the S@pPAN cathode. However, during the 434^th^ cycle, the cell could not be charged to 3.0 V and cell failure occurred.

The cycled cell was disassembled in a glovebox and the dark grey surface of the Li metal was observed (Fig. 5c). The SEM image in Fig. 5d further reveals the Li morphology of rods and dendrites and the severe pulverization. After renewing the Li anode and adding fresh electrolyte, the Li/S@pPAN cell could be further cycled to 800 times without significant capacity fade (Fig. 5b). Thus, it can be concluded that decay of the Li metal anode and electrolyte exhaustion should be the main constraints for the long-term cycling of the Li/S@pPAN battery.

**Fig. 6:**
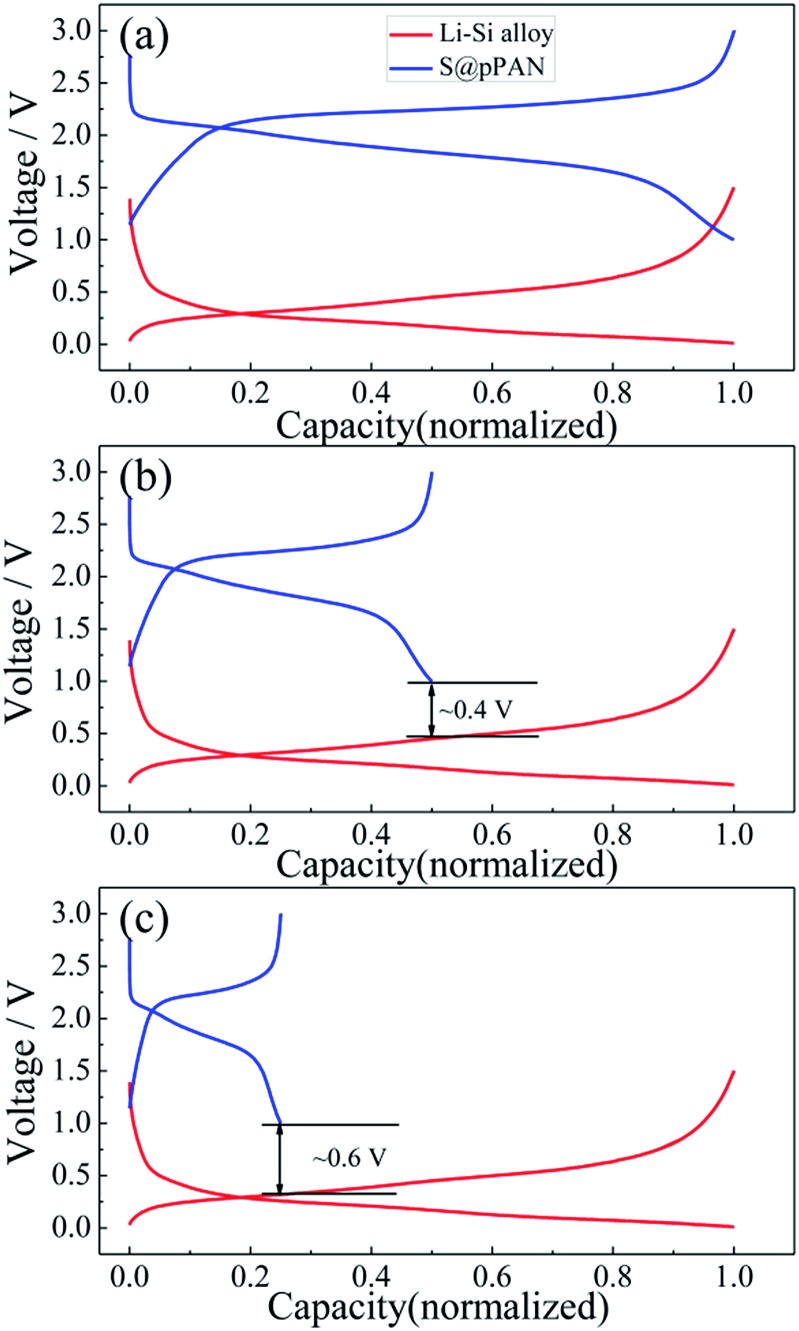
The normalized charge/discharge curves of the Li–S@pPAN cell (1C) and Li–Si alloy electrode (100 mA g^–1^) with anode to cathode capacity ratios of 1 : 1 (a), 2 : 1 (b) and 4 : 1 (c).

Substituting the Li metal anode with an alloy-type anode could be an effective method to address the dendrite growth and other problems. Comparing the normalized charging/discharging curves of S@pPAN and Li–Si alloy electrodes in Fig. 6, it can be inferred that a full cell using the Li–Si anode and S@pPAN cathode could achieve a ∼1.5 V discharging plateau theoretically. In order to increase the cell discharging voltage, the capacity of the anode should be higher than that of the cathode to permit Li extraction from the Li-rich alloy phase (*i.e.* possibly low anode de-lithiation potential). In addition, limiting the de-lithiation depth of the Li–Si electrode can improve its electrochemical reversibility as shown in Fig. S4.† With the capacity ratios of anode to cathode of 2 : 1 and 4 : 1, the terminal discharge voltage of the full cell could reach 0.4 V or 0.6 V (Fig. 6b and c). However, it should be mentioned that a high capacity ratio of anode to cathode will reduce the cell energy density significantly. The practical capacity ratio in the cell design should balance the voltage output, energy density and cycling performance. In the following tests of full cells, the capacity ratio of ∼4 : 1 is adopted for a possibly high voltage output. Cyclic voltammetry (CV) of Li/S@pPAN and Li–Si/S@pPAN cells is compared in Fig. S5.† For the Li/S@pPAN cell, the initial cathodic peak at ∼1.1 V could be ascribed to the activation of S@pPAN and the formation of a solid electrolyte interphase.^35^,^38^,^63^ Thus, the initial coulombic efficiency is ∼75%, which is consistent with the result in Fig. 5a. After the first cycle, the anodic peak at ∼2.4 V and cathodic peak at ∼1.8 V become stable. As for the Li–Si/S@pPAN cell, the initial cathodic peak shifts to ∼1 V, and the following anodic/cathodic peaks are stable at ∼2.2 V/∼1.5 V. The well-overlapped curves for the 2^nd^ and 3^rd^ cycles indicate excellent electrochemical reversibility of the Li–Si/S@pPAN cell.

**Fig. 7:**
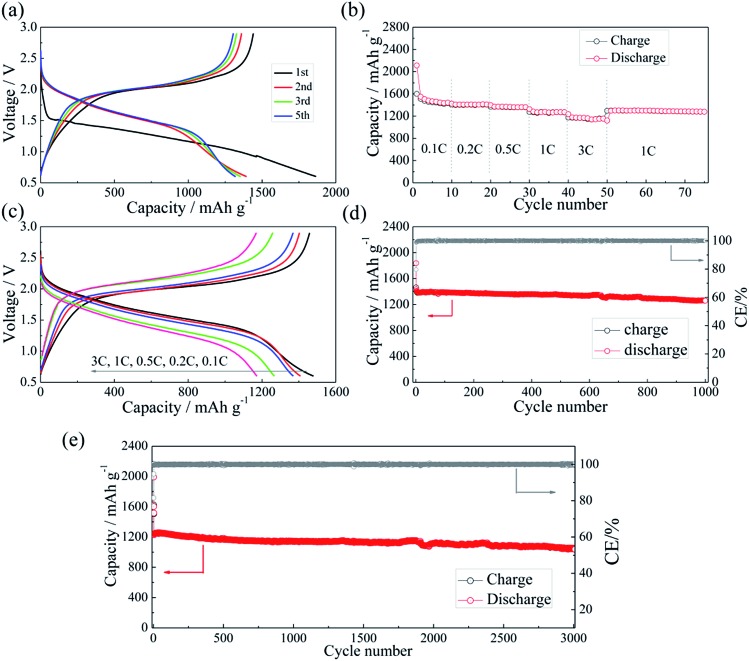
The charge/discharge curves of the Li–Si/S@pPAN cell from 0.6 V to 2.8 V at 1C (a), the rate capability (b and c) and the cycling performance of the full cell at 1C (d) and 3C (e).

The half-cell test shows that the initial charging capacity of the Li–Si anode is larger than its discharge capacity (Fig. S6†). The excess capacity could compensate for the capacity loss of S@pPAN during the initial cycle. Fig. 7a shows the charge/discharge curves of the Li–Si/S@pPAN cell for the initial 5 cycles at 1C (1C = 1675 mA g^–1^) from 0.6 V to 2.8 V. The 1^st^ cycle efficiency is 76.3%, slightly higher than that of the Li/S@pPAN cell (75.3%). The first discharge plateau is at ∼1.25 V, slightly lower than that of the Li/S@pPAN cell. During the following cycles, the discharge plateau shifts to a stable plateau at ∼1.7 V. Thus, a lithium ion cell system with lithium ions shuttling between the anode and cathode has been successfully constructed. As shown in Fig. 7b, the Li–Si/S@pPAN cell possesses exceptional rate capability. When the current rate rises from 0.1C to 3C, the capacity declines from *ca.* 1450 to *ca.* 1170 mA h g^–1^ with a loss of only 19.3%. Moreover, the voltage polarization of the cell is acceptable even with a high rate of 3C (Fig. 7c). The cycling performance of the Li–Si/S@pPAN cell at 1C is shown in Fig. 7d. The initial discharge capacity is 1398 mA h g^–1^, and after 1000 cycles it is 1258 mA h g^–1^, corresponding to a capacity retention of ∼90%. Fig. 7e further exhibits the long-term cycling behavior at 3C. Even after 3000 cycles a capacity of 1075 mA h g^–1^ can be retained with an average fading rate of 0.03% per cycle. It should be mentioned that the coulombic efficiency approaches 99.8% except the first cycle for both 1C and 3C. The high reversibility of the Li–Si/S@pPAN cell should be ascribed to the stable S@pPAN and Li–Si electrode structures and their respective interfacial properties. The Li–Si/S@pPAN cell cycled 1000 times at 1C was disassembled in a glovebox. After washing with DMC and natural drying, it could be noticed that the Li–Si anode retained its good integrity without any pulverization, as shown in Fig. S7a.† The morphology of the cycled anode was further observed with SEM equipment. Fig. S7b and c† demonstrate the smooth electrode surface without obvious cracks and dissociated large particles. High loading sulfur electrodes (∼4.5 mA h cm^–2^) have also been tested in combination with Li metal and Li–Si alloy anodes (∼160 μm thickness, ∼9 mA h cm^–2^). Fig. S8† shows that the Li–Si/S@pPAN cell can retain a discharge capacity of ∼4.3 mA h cm^–2^ at 0.5C, but it encountered a short circuit after 113 cycles because of the existence of Li dendrites. After renewing the Li metal anode, it could be brought back to the normal state (Fig. S8b†). However, when paired with a Li–Si alloy anode, the S@pPAN cathode could be cycled more than 200 times with a capacity fading rate of 0.13% per cycle (Fig. S9b†).

The Nyquist plots of Li/S@pPAN and Li–Si/S@pPAN cells in the charging state after different cycles at 0.5C are shown in Fig. S10.† For the Li/S@pPAN cell, two semicircles emerged in the high frequency region after the first cycle and gradually enlarged during the cycling. They could be mainly attributed to the resistances of the SEI and interfacial reaction on the Li metal (Fig. S10a†). In contrast, for the Li–Si/S@pPAN cell, the semicircles are much smaller and relatively stable during cycling (Fig. S10b†), indicating low and stable interfacial resistances during cycling.

## Conclusions

In summary, a stable lithium-rich Li–Si anode supported by a CNF matrix has been successfully prepared and applied in a lithium-ion–sulfur battery system. Compared with a lithium metal anode, it exhibits no pulverization and no dendrite formation during long cycling. The Li–Si/S@pPAN battery exhibits excellent cycling performance with a negligible capacity fading rate of 0.01% per cycle for 1000 cycles at 1C and 0.03% per cycle for 3000 cycles at 3C. The flexible carbon matrix with its content of 40 wt% buffers the volume change of lithium-rich alloys and guarantees the integrity and sufficient conductivity of the electrode, while the S@pPAN cathode avoids the shuttle effect in carbonate electrolytes. The synergistic effects of these factors endow the full battery with ultra-long cycle life towards practical application.

## Conflicts of interest

There are no conflicts to declare.

## Supplementary Material

Supplementary information
