# Supplementary material for: A high performance lithium-ion–sulfur battery with a free-standing carbon matrix supported Li-rich alloy anode
Source: Chem Sci. 2018 Sep 24;9(47):8829–35. doi: 10.1039/c8sc02897d (PMC6296295; doi:10.1039/c8sc02897d)
Supplement: Supplementary file 1 [file SC-009-C8SC02897D-s001.pdf]

Electronic Supplementary Material (ESI) for Chemical Science.
This journal is © The Royal Society of Chemistry 2018

Supporting Information

A High Performance Lithium-ion Sulfur Battery with Free-standing Carbon Matrix Supported Li-rich Alloy Anode

Tao Zhang, Min Hong, Jun Yang, Zhixin Xu, Jiulin Wang,* Yongsheng Guo, Chengdu Liang*

Experimental

***Preparation of S@pPAN electrodes*:** The S@pPAN was prepared as previously reported.[1] Sublimed sulfur (AR 99.9%, Adamas) and PAN (Mw=150000, Aldrich) were mixed homogenously and then heated at 300 ^{o}C for 6 hours in N_2. After cooling down to room temperature, the black powder was collected with sulfur content of 44%, determined by element analysis (Perkin Elmer PE 2400 II). The electrodes were fabricated by mixing S@pPAN powder, Super P and polyacrylic acid (PAA) in water with mass ratio of 80:10:10, water as solvent. Then the slurry was coated onto carbon coated aluminum foil and dried in vacuum at 80 ^{o}C overnight. The mass loading of S@pPAN was ~2 mg cm^{-2}. For the high loading electrode (S@pPAN ~7 mg cm^{-2}), the slurry was coated onto a nickel foam. The specific capacities of the cells in this paper are based on the mass of sulfur.

***Preparation of Li-Si alloy electrodes*:** Stable lithium metal powder (SLMP from FMC Lithium), nano silicon powder (30~50 nm, HAOXI Research Nanomaterials, Inc.) were mixed in mortar with pestle for 30 minutes in Ar filled glove box. The mass ratio of SLMP to silicon was 1.5:1. Then the obtained black powder was mixed with carbon nanofibres (CNF,diameter ~50 nm, length ~0.8 μm) with mass ratio of 7:3, 6:4 and 5:5, denoted as LiSi-70, LiSi-60 and LiSi-50, respectively. As illustrated in Figure 1, the mixture was pressed into pellets under ~5 MPa with diameter of 11 mm. As for LiSi-60, two kinds of pellets with thicknesses of ~80 and ~160 μm were prepared. The pellets were stored in the glove box after heat treatment in a sealed steel

vessel at 200 ^{o}C. The CNF electrodes were prepared by casting the slurry of CNF and PAA (mass ratio 8:2) onto copper foil and cut into pieces after drying.

Materials characterization: The morphologies of SLMP, nano silicon powder, CNF, cycled lithium metal foil anode, prepared and cycled Li-Si electrodes were characterized by field emission SEM (Nova NanoSEM 450, FEI Company, USA), and energy-dispersive X-ray spectroscopy (EDS) was also measured to examine the element distribution. X-ray diffractions (XRD) were performed on Bruker D8-Advanced diffractometer. X-ray photo electron spectroscopy (XPS) was obtained on an X-ray photoelectron spectrometer (AXIS ULTRA DLD, Kratos Analytical Ltd., UK) with a monochromatic Al Kα source (1486.6 eV). In order to avoid exposure to air, the electrodes were protected by polyimide tape or sealed in a container and quickly transferred into the equipment.

Electrochemical measurement: CR-2016 type coin cells were assembled in the Ar filled glove box. For Li/S@pPAN and Li-Si/S@pPAN cells, Celgard 2400 separator was adopted soaking the electrolyte of 1 M $LiPF_6$ in ethylene carbonate (EC)/dimethyl carbonate (DMC)/ Fluoroethylene Carbonate (FEC) (4.5:4.5:1 by volume). For the Li-Si electrodes evaluation, lithium metal foil was used as anode and the electrolyte was the same with above. The capacity ratio of anode to cathode was about 4:1, but 2:1 for the high sulfur loading electrode. All the cells were galvanostatically cycled on a LAND battery tester (CT2001A). The specific capacities of full cells with S@pPAN cathode were based on the loading of sulfur, and the specific capacities of Li-Si alloy anode were based on the total mass of the electrode. The cycled lithium metal or Li-Si alloy were disassembled in Ar filled glove box and washed by DMC before characterizations. Cyclic voltammetry (CV) was measured on an electrochemical working station (CHI 760E). The electrochemical impedance spectroscopy (EIS) was measured on an Autolab workstation (PGSTAT302N, Metrohm) in the frequency range from 100 KHz to 0.01 Hz.

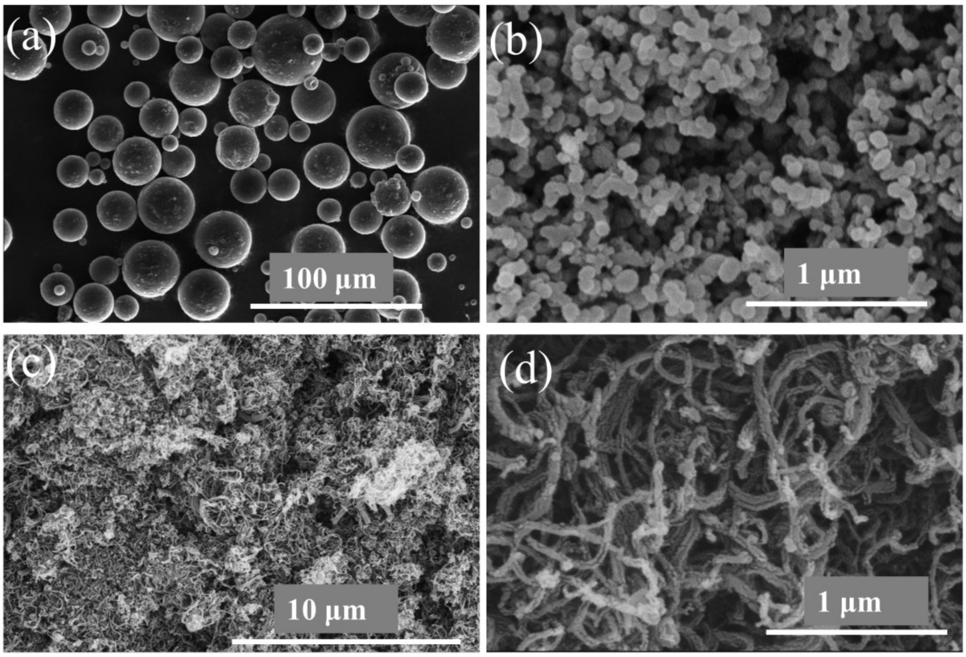

Fig. S1. The SEM images of the SLMP (a), Si powder (b) and CNF (c, d).

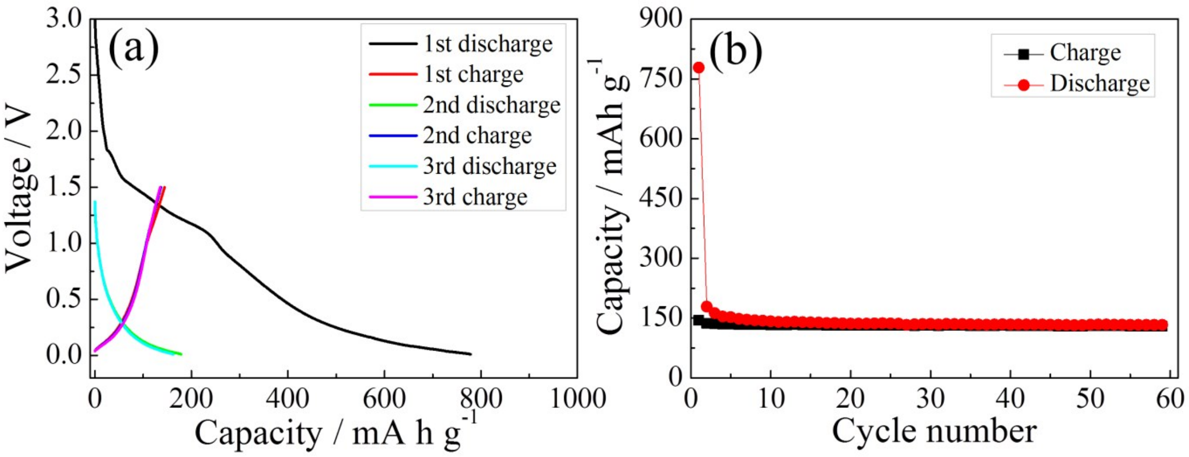

Fig. S2. The initial three discharge/charge cycles of the CNF (a) and the cycling performance (b) at 100 mA g^{-1}.

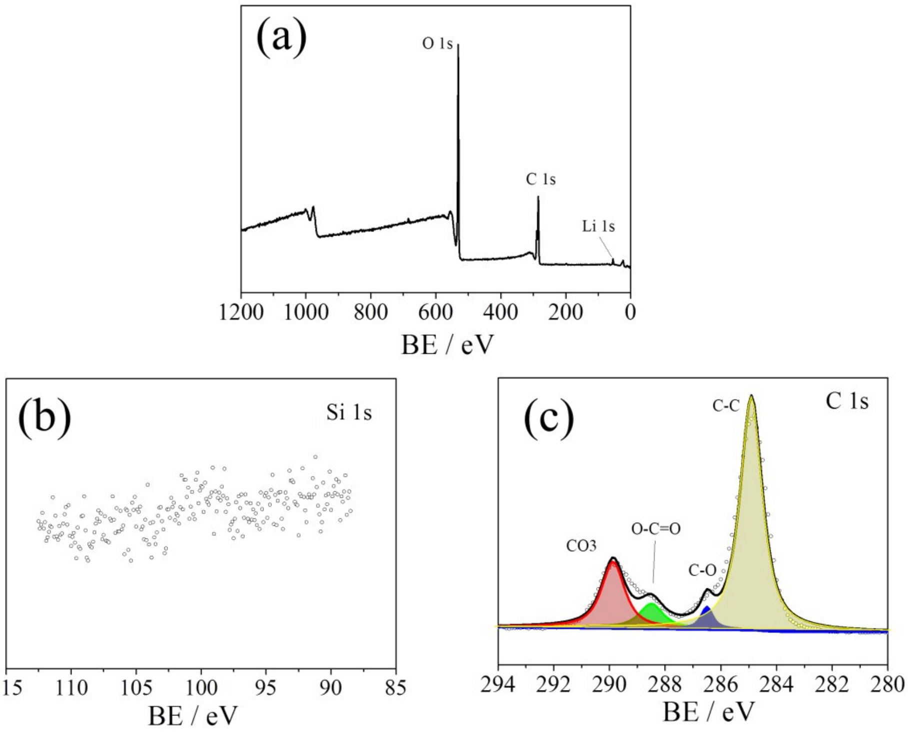

Fig. S3. The XPS spectra of the Li-Si alloy electrode (a), Si 1s (b) and C 1s (c).

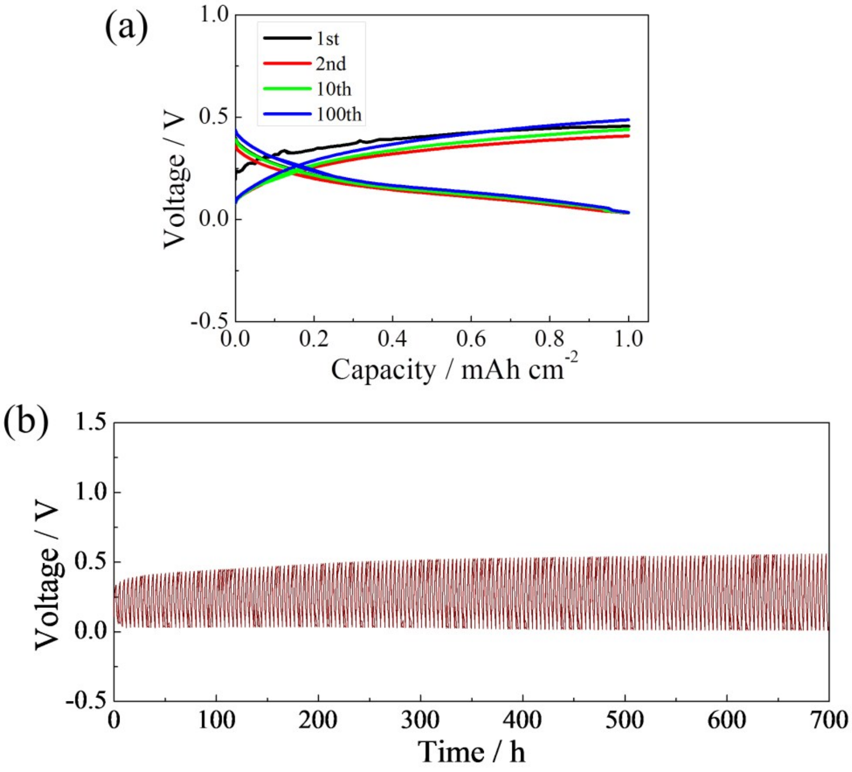

Fig. S4. The charge/discharge curves of the Li-Si alloy electrode with limited capacity of 1 mAh cm^{-2} (a) and cycling performance (b) at 0.5 mA cm^{-2}.

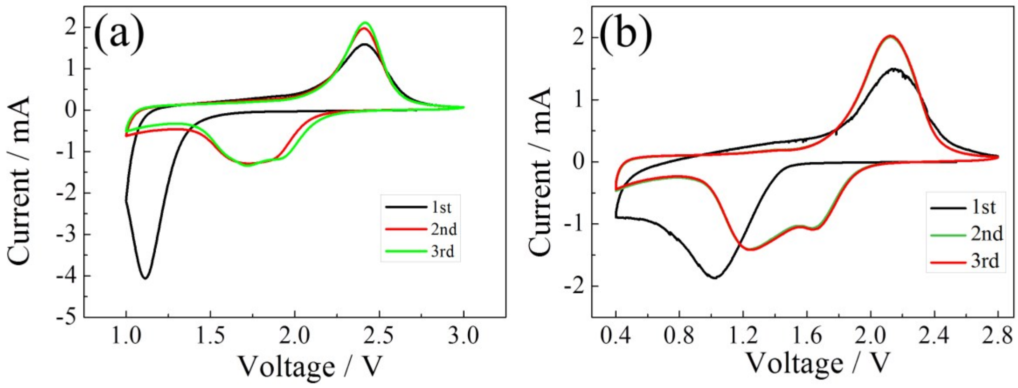

Fig. S5. The CV curves of Li/S@pPAN cell (a: 1~3 V) and Li-Si/S@pPAN cell (b: 0.4~2.8 V) with a scan rate of 0.5 mV s^{-1}.

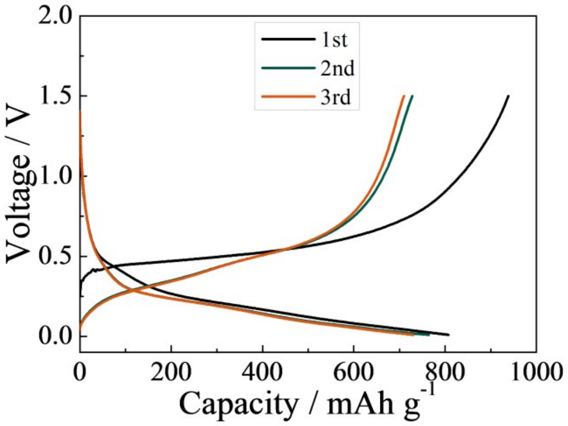

Fig. S6. The charge/discharge curves of the initial three cycles for Li-Si alloy electrode (0.01~1.5 V, 100 mA g^{-1}).

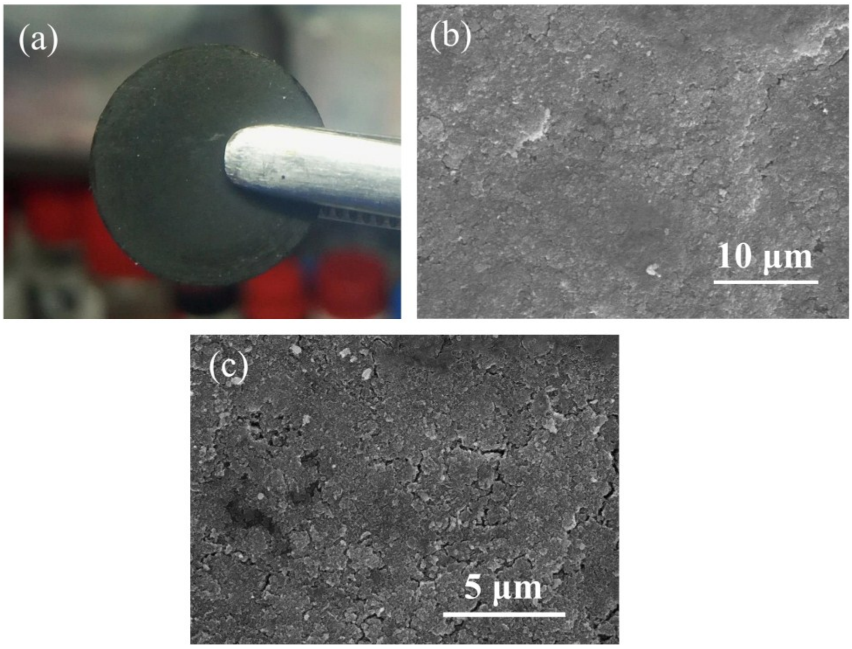

Fig. S7. The photograph image (a) and SEM image (b, c) of the Li-Si alloy electrode cycled for 1000 times at 1C.

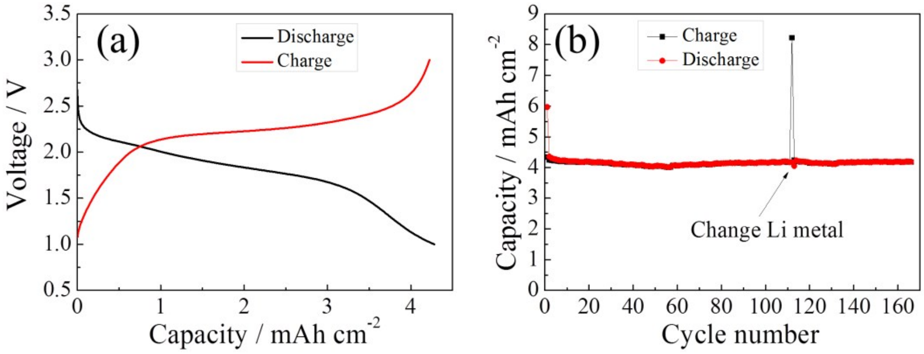

Fig. S8. The typical discharge/charge curve (a) and cycling performance (b) of the Li/S@pPAN cell with high loading sulfur cathode at 0.5C.

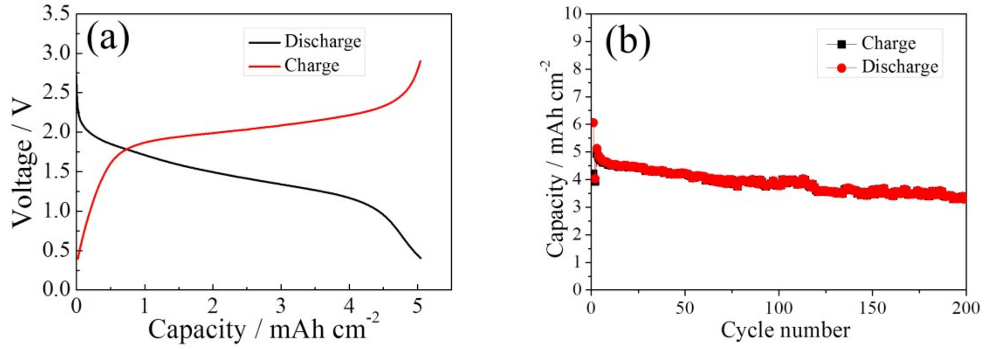

Fig. S9. The typical discharge/charge curve (a) and cycling performance (b) of the Li-Si/S@pPAN cell at 0.5C.

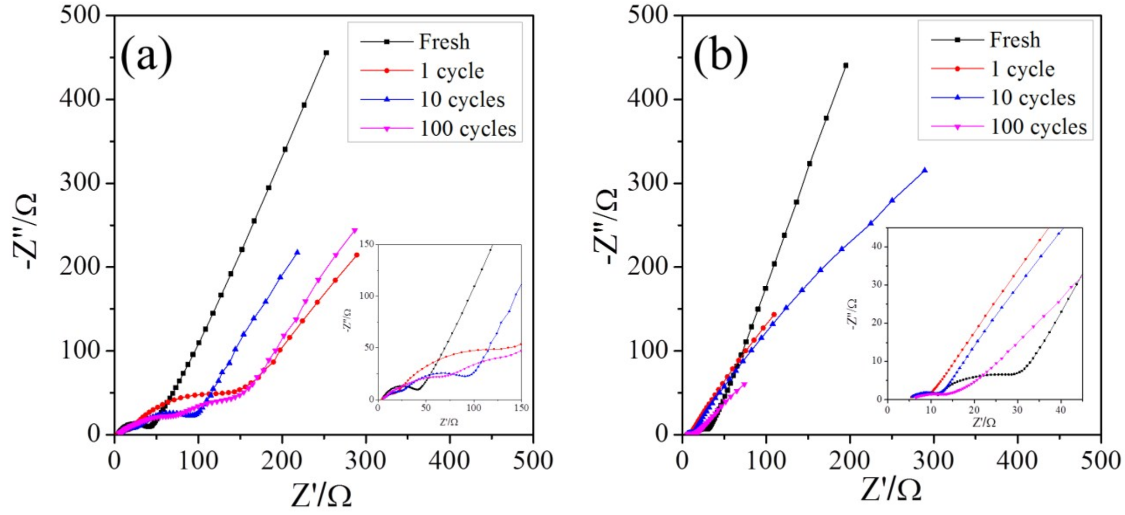

Fig. S10. The Nyquist plots of the Li/S@pPAN cell (a) and Li-Si/S@pPAN cell (b) after different cycles at 0.5C in the charging state.

Reference
[1] Q. Li, H. Yang, L. Xie, J. Yang, Y. Nuli, J. Wang, *Chemical Communications* **2016**, 52, 13479.
